# Association of lncRNA *H19* Gene Polymorphisms with the Occurrence of Hepatocellular Carcinoma

**DOI:** 10.3390/genes10070506

**Published:** 2019-07-04

**Authors:** Edie-Rosmin Wu, Ying-Erh Chou, Yu-Fan Liu, Kuan-Chun Hsueh, Hsiang-Lin Lee, Shun-Fa Yang, Shih-Chi Su

**Affiliations:** 1 Institute of Medicine, Chung Shan Medical University, Taichung 402, Taiwan; 2 Division of General Surgery, Department of Surgery, Lin Shin Hospital, Taichung 402, Taiwan; 3 School of Medicine, Chung Shan Medical University, Taichung 402, Taiwan; 4 Department of Medical Research, Chung Shan Medical University Hospital, Taichung 402, Taiwan; 5 Department of Biomedical Sciences, College of Medicine Sciences and Technology, Chung Shan Medical University, Taichung 402, Taiwan; 6 Division of General Surgery, Department of Surgery, Tungs’ Taichung MetroHarbor Hospital, Taichung 433, Taiwan; 7 Department of Surgery, Chung Shan Medical University Hospital, Taichung 402, Taiwan; 8 Whole-Genome Research Core Laboratory of Human Diseases, Chang Gung Memorial Hospital, Keelung 204, Taiwan; 9 Department of Dermatology, Drug Hypersensitivity Clinical and Research Center, Chang Gung Memorial Hospital, Linkou 24451, Taiwan

**Keywords:** long noncoding RNA, H19, polymorphism, hepatocellular carcinoma

## Abstract

Hepatocellular carcinoma (HCC) is the most common type of primary liver cancer, whose diversified occurrence worldwide indicates a connection between genetic variations among individuals and the predisposition to such neoplasms. Mounting evidence has demonstrated that long non-coding RNA (lncRNA) H19 can have both promotive and inhibitory effects on cancer development, revealing a dual role in tumorigenesis. In this study, the link of *H19* gene polymorphisms to hepatocarcinogenesis was assessed between 359 HCC patients and 1190 cancer-free subjects. We found that heterozygotes for the minor allele of *H19* rs2839698 (T) and rs3741219 (G) were more inclined to develop HCC (OR, 1.291; 95% CI, 1.003–1.661; *p* = 0.047, and OR, 1.361; 95% CI, 1.054–1.758; *p* = 0.018, respectively), whereas homozygotes for the polymorphic allele of rs2107425 (TT) were correlated with a decreased risk of HCC (OR, 0.606; 95% CI, 0.410–0.895; *p* = 0.012). Moreover, patients who bear at least one variant allele (heterozygote or homozygote) of rs3024270 were less prone to develop late-stage tumors (for stage III/IV; OR, 0.566; 95% CI, 0.342–0.937; *p* = 0.027). In addition, carriers of a particular haplotype of three *H19* SNPs tested were more susceptible to HCC. In conclusion, our results indicate an association between *H19* gene polymorphisms and the incidence and progression of liver cancer.

## 1. Introduction

Hepatocellular carcinoma (HCC) is currently the sixth most common type of malignancy with a high death rate and an increasing incidence globally [1]. A heterogeneous occurrence rate of HCC was detected across distinct geographic areas, with the highest rates in Southeast Asia and sub-Saharan Africa [2]. Although approximately 70–90% of HCC occurs within a well-established background of chronic liver diseases [3], liver tumorigenesis is a complex process that is correlated to a variety of risks such as exposure of aflatoxin B, chronic infection with hepatitis B virus (HBV) or hepatitis C virus (HCV), excessive consumption of alcohol and tobacco, iron overload, and diabetes [4,5]. In addition, recent studies have revealed that single-nucleotide polymorphisms (SNPs) are associated with the formation of hepatic neoplasm independently or together with the recognized risk factors in particular ethnic populations [6,7,8]. These findings suggest that an individual’s gene polymorphisms influence oxidative stress, DNA repair, iron metabolism, cell signaling, inflammatory and immune responses, which contribute to the predisposition to hepatocarcinogenesis and partly address the global heterogeneous incidence of HCC.

Current large-scale sequencing studies have revealed that a considerable part of the human genome is transcribed into RNA, but just less than 2% encodes for proteins [9,10]. This has shifted our understanding of functional genomics from messenger RNAs to the noncoding transcriptome, with attention being given to the recognition of an expanding category of long noncoding RNAs (lncRNAs). Arbitrarily described as RNA molecules that are greater than 200 nucleotides and possess no protein-coding potential, lncRNAs exhibit diverse functionality by controlling transcription, translation, and regulation of cellular signaling cascades [11]. In addition to their versatile functionality, lncRNAs are estimated to outnumber the protein-coding genes [9]. Moreover, mounting evidence has implicated an increasing list of lncRNAs in the pathogenesis of a great variety of human disorders [12], including cancer. Extensive transcriptomic investigations have connected the delicate orchestration of lncRNA expression to cancer initiation and poor outcome in various tumors, and surveys of cancer genomes have uncovered a catalogue of functional variants within the lncRNA genes [13,14], highlighting a strong link between tumorigenesis and the modulation of lncRNAs.

H19 is an oncofetal lncRNA that is expressed in the embryo, downregulated at birth and then replenished in tumors [15]. The broad spectrum of H19’s oncogenic actions covers the complex process of tumorigenesis, including translational dysregulation, genomic instability, proliferative imbalance, and metastasis. However, numerous investigations point to the contradictory effects of H19 on tumor development, progression, and treatment [16,17,18,19,20], and indicate the complexity of H19 functionality. In addition, polymorphisms within the *H19* gene in many ethnic populations have been related to the susceptibility to various tumor types, including bladder [21,22], gastric [23], colorectal [24], lung [25], breast [26,27,28], ovarian [29,30], liver [31], bone [32], and oral cancer [33]. Nevertheless, these results provide no consensus regarding the promotive or protective effect of individual *H19* SNPs on cancer risk. Here, we conducted a hypothesis-driven case-control study to assess the correlation of *H19* gene variations with the incidence and clinical parameters of HCC and detected associations of HCC risk with *H19* SNPs and haplotype.

## 2. Materials and Methods

### 2.1. Subjects

This hospital-based study, consisting of 359 patients with HCC and 1190 cancer-free controls accrued from 2006 to 2017, was approved by the institutional review board of Chung Shan Medical University Hospital in Taichung, Taiwan (CSMUH No: CS15099 approved the 20 August 2015). All participants provided informed written consent at enrollment. Diagnosis of all cases was histologically verified, and their clinical stage was assigned at the time of diagnosis according to the TNM staging system of the American Joint Committee on Cancer (AJCC) [34]. Diagnosis of liver cirrhosis was based on liver biopsy, biochemical evidence of liver parenchymal damage with endoscopic esophageal or gastric varices, or abdominal sonography. Clinical features, including liver cirrhosis, the levels of α-fetoprotein (AFP), aspartate aminotransferase (AST), alanine aminotransferase (ALT), tumor staging, tumor size, lymph-node metastasis, distant metastasis, presence of HBV surface antigen (HBsAg), and reactivity with antibody against HCV (anti-HCV), were collected from the chart reviews. Within the same study period, 1190 ethnicity-matched individuals who have neither self-reported history of cancer of any sites nor diagnosed with HCC were enrolled as the controls.

### 2.2. Demographic Data

A survey concerning age, gender, alcohol drinking, and cigarette smoking was collected from each subject. Having up to an average of more than 2 drinks per day was considered alcohol consumption. Current smoking of at least one cigarette per day during the latest three months was considered a persistent smoking habit.

### 2.3. Genotyping

We used QIAamp DNA blood mini kits (Qiagen, Valencia, CA, USA) to isolate genomic DNA. TaqMan assay with an ABI StepOne™ Real-Time PCR System (Applied Biosystems, Foster City, CA, USA) was used to evaluate allelic discriminations of five *H19* SNPs (rs217727, rs2107425, rs2839698, rs3024270, and rs3741219). Genotypes were analyzed with SDS version 3.0 software (Applied Biosystems).

### 2.4. Predicting the Structure of H19/miRNA Duplex

The interaction between H19 and miRNA targets was predicted by the RNAhybrid algorithm, which determines the most favorable hybridization site between two RNA sequences [35].

### 2.5. Statistical Analysis

The Hardy-Weinberg equilibrium was evaluated by using a goodness-of-fit v2 test for biallelic markers. Differences in demographic characteristics between healthy controls and HCC patients were compared by using the Mann–Whitney U test and Fisher’s exact test. The adjusted odds ratios (AORs) with their 95% confidence intervals (CIs) for the association between genotype frequencies and the risk of HCC plus clinicopathological characteristics were assessed by multiple logistic regression models after controlling for other covariates. The haplotype-based analysis was performed using the Phase program [36]. A *p*-value < 0.05 was considered significant. The data were analyzed by using SAS statistical software (Version 9.1, 2005; SAS Institute Inc., Cary, NC, USA).

## 3. Results

### 3.1. Characteristics of Subjects

Since various risk factors, such as age, gender, alcohol consumption, and tobacco use have been demonstrated to contribute to the etiology and pathogenesis of liver cancer [37,38], we first compared the demographic information of 359 HCC patients with that from 1190 normal controls (Table 1). As no difference in the ratio of males to females was achieved between the case and control group (*p* = 0.678), subjects with advancing age were more prone to develop HCC with the average age of patients at onset of HCC in this study being 62.9 ± 11.5. In addition, we observed that alcohol consumption, but not tobacco use (*p* = 0.762), tended to elevate the risk of developing HCC.

### 3.2. Association of H19 Gene Polymorphisms with HCC

**Figure 1:**
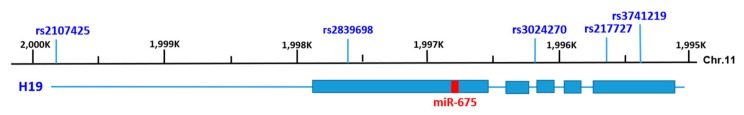
Genomic structure of human H19 and locations of the single-nucleotide polymorphisms (SNPs) examined in this study. The genome structure of human lncRNA H19 is within the region of 1995 K–2000 K bp of chromosome 11 for the GRCh38.p12 primary assembly. H19 contains 5 exons as represented by boxes in blue and flanking introns shown by lines. miR-675, which is embedded in the 1st exon of H19, is indicated by the red box. The five SNPs tested, rs2107425 (in the promoter region), rs2839698 (exonic), rs3024270 (intronic), rs217727 (exonic) and rs3741219 (exonic), are shown above the scale.

To determine whether H19 gene polymorphism was associated with the risk of HCC, genotype frequencies of five H19 SNPs (rs217727, rs2107425, rs2839698, rs3024270, and rs3741219) (Figure 1) and their association with the susceptibility to liver cancer were assessed (Table 2). For five SNPs tested, no deviation (p > 0.05) from Hardy-Weinberg equilibrium was detected in either the case or control group. We utilized AOR (with 95% CI), which was estimated by multiple logistic regression models after adjustment for two potential confounders, age and alcohol consumption, together with OR (with 95% CI) in each comparison. Among the loci studied, heterozygotes for the minor allele of H19 rs2839698 (T) and rs3741219 (G) were more inclined to have HCC with the OR being 1.291 (95% CI, 1.003–1.661; p = 0.047), and 1.361 (95% CI, 1.054–1.758; p = 0.018), respectively. Although adjusted for age and alcohol use, their association with a predisposition to liver cancer (AOR, 1.353; 95% CI, 1.038–1.765; p = 0.026, for rs2839698; AOR, 1.429; 95% CI, 1.092–1.817; p = 0.009, for rs3741219) was further strengthened. Intriguingly, we detected an association between the homozygous genotype for the polymorphic allele of rs2107425 (TT) and a decreased risk of HCC (OR, 0.606; 95% CI, 0.410–0.895; p = 0.012 and AOR, 0.616; 95% CI, 0.409–0.926; p = 0.02). Nevertheless, no difference in genotypic frequencies for rs217727 and rs3024270 individually was identified between the two study groups.

### 3.3. Correlation between Polymorphic Genotypes of H19 and Clinical Status of HCC

Since H19 gene polymorphisms were found to be correlated with susceptibility to liver cancer, the relationship between the H19 gene variations and clinicopathologic characteristics of HCC patients was also evaluated in this study. We observed that patients who possess at least one variant allele (heterozygote or homozygote for the minor allele) of rs3024270, rather than rs2107425, (Table 3) were less prone to develop late-stage tumors (for stage III/IV; OR, 0.566; 95% CI, 0.342–0.937; p = 0.027, and AOR, 0.564; 95% CI, 0.340–0.935; p = 0.026). However, none of the SNPs tested was found to be correlated with the levels of serological markers of HCC, including α-fetoprotein (AFP), alanine transaminase (ALT), and aspartate transaminase (AST) (Table 4). These data suggest that different genotypes of different SNPs within H19 gene may exert additional effects on tumor suppression besides their oncogenic capacities.

**Figure 2:**
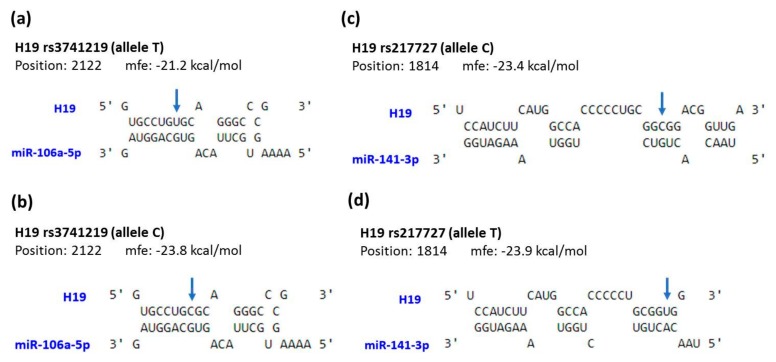
Prediction of potential binding between *H19* and its interacting microRNAs. The structure exhibits hybridization between miR-106a and *H19* with rs3741219 allele T (**a**) or C (**b**), as well as between miR-141 and H19 with rs217727 allele C (**c**) or T (**d**). The positions of exonic SNPs are indicated by blue arrows. mfe, minimum free energy.

In addition, to provide a preliminary assessment of how the change in the exonic sequences affects the putative function of H19, a bioinformatic analysis for the interaction of H19 with its microRNA targets was performed. H19 was previously reported to act as a natural sponge for many microRNAs [39,40,41]. Here, we showed that transcripts of H19 derived from distinct genotypes for three exonic SNPs examined in this study had different affinities and formed different secondary structures with miR-106a and miR-141 (Figure 2).

### 3.4. Association between H19 Haplotypes and HCC

The link between *H19* gene haplotypes and the risk of HCC was also investigated. The frequency distributions of seven *H19* rs2107425, rs2839698, and rs3741219 haplotypes are shown in Table 5, with the most common haplotype in the controls (TCA) being selected as the reference. We found that a specific haplotype of *H19* (CTG) was significantly associated with increased susceptibility to HCC (OR, 1.237; 95% CI, 1.015–1.507; *p* = 0.035; AOR, 1.240; 95% CI, 1.008–1.526; *p* = 0.042), further suggesting a genetic predisposition of *H19* to liver cancer.

## 4. Discussion

The initiation and progression of liver cancer is a series of complicated actions influenced by both inherited and external factors. In this study, we found that gene variations of *H19* rs2839698 and rs3741219 increase the predisposition to HCC, whereas rs2107425 and rs3024270 are associated with a decreased risk of HCC occurrence and developing advanced tumors, respectively. Our data reveal the complexity of *H19* gene variations in regulating hepatocarcinogenesis.

Even though the significance of H19 in cancer has been recognized for years, its exact role in tumorigenesis is still a subject of controversy as both oncogenic and oncostatic effects have been demonstrated [42]. Moreover, it has been proposed that the functionality of H19 in liver cancer is seemingly much more intricate than that in other types of tumors [42,43] because of the highly heterogeneous etiology. This involves a complex interplay between genetic alterations and inflammatory conditions associated with viral hepatitis as well as with alcoholic and non-alcoholic steatohepatitis [44]. In this study, we found that two exonic SNPs of *H19*, rs2839698 and rs3741219, are correlated with elevated susceptibility to liver cancer (Table 2). *H19* gene consists of five exons, and rs2839698 and rs3741219 are located within the 1st and 5th exon, respectively. Largely consistent with findings from other reports [23,24,31,45], we also detected an association between rs2839698 and an increased risk of HCC. However, the rs2839698 polymorphism has been shown to exhibit the opposite associations for cancer risk in a population in the Netherlands [21]. It is conceivable that variations in the exonic region of *H19* may alter its conserved secondary structure or sequence complementarity to its target genes (chromatin or mRNA), thereby modifying its binding affinity to the interacting partners (Figure 2). Supporting this notion, a previous bioinformatic analysis has shown that rs2839698 may change *H19*’s crucial folding structures and targeted microRNAs [24]. In addition, we, for the first time, observed an association between increased risk of HCC and another exonic SNP of *H19*, rs3741219 (Table 2). Of note, the region of *H19* loci where rs3741219 resides expresses another antisense transcript named the H19 opposite tumor suppressor (HOTS) [46]. The HOTS transcript encodes for a nucleolar protein in primates but lacks an open reading frame in mice [47]. It is demonstrated that HOTS acts as a tumor suppressor in vivo and that the levels of HOTS and H19 appear to be uncoordinated [47]. Such complexity may in part account for the discrepancy in the association between individual *H19* SNP and distinct cancer types.

While exonic SNPs probably alter H19’s conserved secondary structure or sequence complementarity to the target genes, upstream or intronic SNPs are more likely involved in transcriptional regulation and alternative splicing of *H19* transcript. Unlike the two exonic SNPs mentioned above, we found that an upstream SNP of *H19*, rs2107425, is associated with a decreased risk of HCC (Table 2). Such a protective effect of rs2107425 against cancer development is in concordance with the findings of two recent studies using meta-analysis [45,48]. rs2107425 is located in the differentially methylated region (DMR) of *H19*, which is upstream of the transcription start site and acts as a part of a methylation-sensitive insulator. It has been reported that H19 DMR hypermethylation is associated with loss of imprinting of the *IGF2* gene, which often results in IGF2 overexpression [49]. The results from our and others’ investigations suggest that rs2107425 gene polymorphism may regulate the expression of *H19* and its co-expressed genes through an epigenetic mechanism to affect the outcome of this devastating disease. In addition, we found that patients possessing at least one polymorphic allele of an intronic SNP, rs3024270, have a deceased risk of developing advanced HCC tumors (Table 3). Similarly, in patients with invasive bladder cancer, it has been shown that homozygous carriers of the minor allele of rs3024270 may have a better prognosis [50]. Taken together, we show that two exonic SNPs of *H19*, rs2839698 and rs3741219, render individuals more susceptible to HCC, whereas upstream or intronic SNPs of *H19* (rs2107425 and rs3024270) exhibit protective effects on the development of liver cancer, revealing a functional complexity of *H19* gene polymorphism in governing hepatocarcinogenesis.

Our results reveal an effect of *H19* gene polymorphisms on the incidence and progression of HCC; nevertheless, extra work is needed to address several limitations of this study. One is that the impacts of environmental factors on the risk of liver cancer may be underestimated owing to a lack of cohort stratification based on the levels of alcohol consumption. Another weakness is that the high degree of heterogeneity in the disease severity or HCC-associated clinical manifestations, such as viral hepatitis as well as with alcoholic and non-alcoholic steatohepatitis within HCC patients may result in distinct findings regarding the link between *H19* gene polymorphisms and liver tumorigenesis. In addition, although numerous non-coding variants within lncRNA genes were identified as expression quantitative trait loci [51], we failed to further prove that the upstream or intronic SNPs examined in this study affect the expression of H19 and its co-expressed genes due to a lack of expression data for H19 and its targets in our cohort. Furthermore, the genetic association detected in the present investigation might be limited to unique ethnic group unless replication experiments are performed.

## 5. Conclusions

In conclusion, data from our present investigation demonstrated that gene polymorphisms (rs2839698 and rs3741219) and a specific haplotype of *H19* confer an increased susceptibility to HCC. However, an inverse association between the other two SNPs, rs2107425 and rs3024270 and the occurrence and progression of HCC was observed. These results reveal the intricacy of H19 functions and the dual role of *H19* polymorphisms in the development of hepatic tumors.

## Figures and Tables

**Table 1:** The distributions of demographical characteristics in 1190 controls and 359 patients with hepatocellular carcinoma (HCC).

Variable	Controls (N = 1190)	Patients (N = 359)	*p*-Value
Age (yrs)	59.4 ± 7.1	62.9 ± 11.5	*p* < 0.001 *
Gender			
Male	835 (70.2%)	256 (71.3%)	
Female	355 (29.8%)	103 (28.7%)	*p* = 0.678
Cigarette smoking			
No	720 (60.5%)	214 (59.6%)	
Yes	470 (39.5%)	145 (40.4%)	*p* = 0.762
Alcohol drinking			
No	1022 (85.9%)	227 (63.2%)	
Yes	168 (14.1%)	132 (36.8%)	*p* < 0.001 *
HBsAg			
Negative		208 (57.9%)	
Positive		151 (42.1%)	
Anti-HCV			
Negative		193 (53.8%)	
Positive		166 (46.2%)	
Stage			
I+II		249 (69.4%)	
III+IV		110 (30.6%)	
Tumor T status			
T1+T2		252 (70.2%)	
T3+T4		107 (29.8%)	
Lymph node status			
N0		349 (97.2%)	
N1+N2+N3		10 (2.8%)	
Metastasis			
M0		341 (95.0%)	
M1		18 (5.0%)	
Child-Pugh grade			
A		281 (78.3%)	
B or C		78 (21.7%)	
Liver cirrhosis			
Negative		65 (18.1%)	
Positive		294 (81.9%)	

Mann-Whitney U test or Fisher’s exact test was used between healthy controls and patients with HCC. * *p*-value < 0.05 as statistically significant.

**Table 2:** Genotypic frequency of *H19* SNP in HCC and normal controls.

Variable	Controls (N = 1190) (%)	Patients (N = 359) (%)	OR (95% CI)	AOR (95% CI) ^a^
**rs217727**				
CC	495 (41.6%)	154 (42.9%)	1.000 (reference)	1.000 (reference)
CT	539 (45.3%)	170 (47.3%)	1.014 (0.790–1.302)	1.044 (0.802–1.358)
TT	156 (13.1%)	35 (9.8%)	0.721 (0.479–1.085)	0.727 (0.474–1.116)
**rs2107425**				
CC	422 (35.5%)	134 (37.3%)	1.000 (reference)	1.000 (reference)
CT	560 (47.0%)	185 (51.5%)	1.040 (0.806–1.344)	1.041 (0.795–1.362)
TT	208 (17.5%)	40 (11.2%)	0.606 (0.410–0.895) ^b^	0.616 (0.409–0.926) ^e^
**rs2839698**				
CC	532 (44.7%)	140 (39.0%)	1.000 (reference)	1.000 (reference)
CT	524 (44.0%)	178 (49.6%)	1.291 (1.003–1.661) ^c^	1.353 (1.038–1.765) ^f^
TT	134 (11.3%)	41 (11.4%)	1.163 (0.782–1.728)	1.165 (0.769–1.763)
**rs3024270**				
CC	334 (28.1%)	87 (24.2%)	1.000 (reference)	1.000 (reference)
GC	593 (49.8%)	187 (52.1%)	1.211 (0.908–1.614)	1.237 (0.915–1.672)
GG	263 (22.1%)	85 (23.7%)	1.241 (0.883–1.743)	1.237 (0.866–1.768)
**rs3741219**				
AA	517 (43.5%)	129 (35.9%)	1.000 (reference)	1.000 (reference)
GA	536 (45.0%)	182 (50.7%)	1.361 (1.054–1.758) ^d^	1.429 (1.092–1.817) ^g^
GG	137 (11.5%)	48 (13.4%)	1.404 (0.959–2.056)	1.368 (0.918–2.039)

^a^ Adjusted for the effects of age and alcohol drinking; ^b^ *p* = 0.012; ^c^ *p* = 0.047; ^d^ *p* = 0.018; ^e^ *p* = 0.020; ^f^ *p* = 0.026; ^g^ *p* = 0.009.

**Table 3:** Odds ratio (OR) and 95% confidence interval (CI) of clinical status and *H19* rs3024270 genotypic frequencies in 359 HCC patients.

Variable	Genotypic Frequencies			
	CC (N = 87)	GC + GG (N = 272)	OR (95% CI)	AOR (95% CI) ^a^
Clinical Stage				
Stage I/II	52 (59.8%)	197 (72.4%)	1.00	1.00
Stage III/IV	35 (40.2%)	75 (27.6%)	0.566 (0.342–0.937) ^b^	0.564 (0.340–0.935) ^c^
Tumor Size				
≤T2	54 (63.3%)	198 (72.8%)	1.00	1.00
>T2	33 (26.7%)	74 (27.2%)	0.612 (0.368–1.017)	0.611 (0.367–1.017)
Lymph Node Metastasis				
No	83 (95.4%)	266 (97.8%)	1.00	1.00
Yes	4 (4.6%)	6 (2.2%)	0.468 (0.129–1.698)	0.484 (0.132–1.778)
Distant Metastasis				
No	81 (93.1%)	260 (95.6%)	1.00	1.00
Yes	6 (6.9%)	12 (4.4%)	0.623 (0.227–1.713)	0.624 (0.226–1.723)
Vascular Invasion				
No	68 (78.2%)	234 (86.0%)	1.00	1.00
Yes	19 (21.8%)	38 (14.0%)	0.581 (0.315–1.073)	0.572 (0.309–1.060)
Child-Pugh Grade				
A	65 (74.7%)	216 (79.4%)	1.00	1.00
B or C	22 (25.3%)	56 (20.6%)	0.766 (0.435–1.349)	0.766 (0.435–1.351)
Liver Cirrhosis				
Negative	16 (18.4%)	49 (18.0%)	1.00	1.00
Positive	71 (81.6%)	223 (82.0%)	1.026 (0.549–1.915)	1.013 (0.539–1.902)

The ORs analyzed by their 95% CIs were estimated by logistic regression models, >T2: multiple tumor more than 5 cm or tumor involving a major branch of the portal or hepatic vein(s), * *p*-value < 0.05 as statistically significant, ^a^ Adjusted for the effects of age and alcohol drinking, ^b^ *p* = 0.027, ^c^ *p* = 0.026, ^d^ *p* = 0.037.

**Table 4:** Association between *H19* genotypic frequencies and the HCC laboratory findings.

Characteristic	α-Fetoprotein ^a^ (ng/mL)	AST ^a^ (IU/L)	ALT ^a^ (IU/L)	AST/ALT ^a^ Ratio
**rs217727**				
CC	464.9 ± 201.5	52.6 ± 5.9	45.1 ± 3.6	1.2 ± 0.1
CT + TT	932.2 ± 301.0	47.3 ± 4.5	46.0 ± 4.2	1.2 ± 0.1
*p*-value	0.197	0.480	0.867	0.880
*p*-value ^b^	0.234	0.468	0.873	0.884
**rs2107425**				
CC	556.9 ± 236.3	45.9 ± 3.4	42.0 ± 3.1	1.2 ± 0.02
CT + TT	836.9 ± 272.6	51.5 ± 5.3	47.6 ± 4.1	1.2 ± 0.02
*p*-value	0.438	0.367	0.277	0.925
*p*-value ^b^	0.489	0.446	0.347	0.930
**rs2839698**				
CC	749.2 ± 296.7	54.9 ± 7.6	47.3 ± 5.5	1.2 ± 0.02
CT + TT	726.6 ± 257.1	45.4 ± 2.6	44.3 ± 2.8	1.2 ± 0.03
*p*-value	0.954	0.234	0.618	0.255
*p*-value ^b^	0.954	0.185	0.593	0.277
**rs3024270**				
CC	660.7 ± 325.5	52.1 ± 9.2	49.7 ± 8.1	1.2 ± 0.02
CG + GG	764.6 ± 237.5	48.5 ± 3.6	44.1 ± 2.5	1.2 ± 0.03
*p*-value	0.797	0.656	0.512	0.270
*p*-value ^b^	0.812	0.653	0.385	0.385
**rs3741219**				
AA	590.3 ± 246.5	52.7 ± 7.5	45.7 ± 5.5	1.2 ± 0.02
GA + GG	840.9 ± 282.8	47.2 ± 3.1	45.5 ± 3.0	1.2 ± 0.03
*p*-value	0.525	0.498	0.972	0.302
*p*-value ^b^	0.524	0.450	0.970	0.329

Mann-Whitney U test was used between two groups. ^a^ Mean ± S.E. ^b^ Adjusted age and alcohol drinking.

**Table 5:** Frequency of *H19* haplotypes in HCC patients and control subjects.

Haplotype Block			Controls	Patients		
rs2107425 C/T	rs2839698 C/T	rs3741219 A/G	N = 2380	N = 718	OR (95% CI)	AOR (95% CI) ^a^
T	C	A	946 (39.8%)	260 (36.2%)	1.000 (reference)	1.000 (reference)
C	T	G	750 (31.5%)	255 (35.5%)	1.237 (1.015–1.507) ^b^	1.240 (1.008–1.526) ^c^
C	T	A	582 (24.5%)	175 (24.4%)	1.094 (0.880–1.360)	1.074 (0.855–1.350)
C	C	G	43 (1.8%)	18 (2.5%)	1.523 (0.864–2.685)	1.404 (0.770–2.559)
C	T	A	29 (1.2%)	5 (0.7%)	0.627 (0.240–1.637)	0.734 (0.271–1.989)
T	C	G	17 (0.7%)	5 (0.7%)	1.070 (0.391–2.928)	1.220 (0.432–3.446)
T	T	A	13 (0.5%)	0 (0.0%)	-	-

^a^ Adjusting for the effects of age and alcohol drinking; ^b^ *p* = 0.035; ^c^ *p* = 0.042.
